# Effect of Ultrasonic Shot Peening and Laser Shock Peening on the Microstructure and Microhardness of IN738LC Alloys

**DOI:** 10.3390/ma16051802

**Published:** 2023-02-22

**Authors:** Sijia Liu, Youngdae Kim, Jinesung Jung, Seongguk Bae, Sungho Jeong, Keesam Shin

**Affiliations:** 1 School of Materials Science and Engineering, Changwon National University, Changwon 51140, Republic of Korea; 2 Power Generation Technology Laboratory, KEPCO Research Institute, Daejeon 34056, Republic of Korea; 3 School of Mechanical Engineering, Gwangju Institute of Science and Technology, Gwangju 61005, Republic of Korea

**Keywords:** laser shock peening, ultrasonic shot peening, IN738LC, microstructure

## Abstract

IN738LC is a conventional-cast Ni-based superalloy intended for power generation and aerospace applications. Typically, ultrasonic shot peening (USP) and laser shock peening (LSP) are utilized to enhance cracking, creep, and fatigue resistance. In this study, the optimal process parameters for USP and LSP were established by observing the microstructure and measuring the microhardness of the near-surface region of IN738LC alloys. The LSP impact region (modification depth) was approximately 2500 μm, which was much higher than the USP impact depth of 600 μm. The observation of the microstructural modification and resulting strengthening mechanism revealed that the build-up of dislocations upon peening with plastic deformation was crucial for alloy strengthening in both methods. In contrast, significant strengthening via γ′ shearing was observed only in the USP-treated alloys.

## 1. Introduction

IN738LC, patented by Inconel Corporation in 1969 [1], is a commonly used conventionally-cast Ni-based superalloy created and developed for use in hot-section components for turbines, such as rotor blade stages 1, 2, and 3 [2]. Due to the optimal concentrations of essential elements (Cr, Mo, Co, Al, W, and Ta) [3,4], it is resistant to corrosion/oxidation at temperatures of up to 950 °C in high-stress situations.

However, during operation, high-temperature creep is a primary cause of failure. Mechanical vibration and damage from foreign objects cause fatigue, an issue for all blade materials. In addition, the collision of debris with the surface can generate microcracks, leading to failure/rupture [5,6]. Therefore, ensuring the integrity of the material, such as its smoothness, is of interest to manufacturers of turbine blades. Poor surface and microstructural integrity have been the focus of study to extend the life of turbine blades. Ultrasonic shot peening (USP) and laser shock peening (LSP) are effective methods for enhancing surface integrity and lifetime extension.

USP is a surface treatment process where the metallic sample surface is hit with hard balls to generate compressive stress and defects to increase fatigue resistance [7]. In contrast, LSP is a process where metallic materials are irradiated with a high-energy laser to introduce defects, such as twinning and compressive stress, to impart fatigue resistance [8]. The improvement of mechanical properties following LSP treatment, including hardness [9,10,11,12,13,14,15,16,17,18,19], wear resistance [20], corrosion resistance [21,22], elastic modulus [18], and yield strength [23], has been investigated. Wang et al. [9] reported that dislocation multiplication and interweaving inhibited dislocation slip and movement, generating advantageous features. Producing high-density dislocations is an important and effective method for improving strength. The nucleation, multiplication, and intertwining of new dislocations during the peening process change the preceding slip direction and make slipping more difficult, effectively improving the mechanical properties [9,24,25].

USP and LSP are two distinct approaches that have been explored extensively for their distinct benefits. For instance, USP promotes surface deformation and refines the grains of the surface layer, but its depth is less than that of LSP. Utilizing both USP and LSP is advantageous, as more mechanisms are available for microstructural evolution. USP is inexpensive and simple to adapt and administer but must be carried out under optimum conditions, since elongated USP treatment may lead to recrystallization and softening with surface contamination and roughening via collisions. In contrast, LSP modifies the microstructure to a depth as deep as a few mm [15], but the procedure is time-consuming and laser burns often occur.

While USP and LSP are commonly applied industrially to improve the surface properties of alloys, their comparative microstructural studies on the same material are rare. In addition, research on the microstructural modification by LSP is vague and insufficient compared to USP.

This is a very rare and valuable comparative study about the two techniques applied on IN738LC to determine their effects on the microstructure and mechanical properties of the alloy, to determine the optimum processing conditions and strengthening mechanisms, i.e., the characteristics and application of USP and LSP for high-temperature components, such as turbine blades.

## 2. Materials and Methods

### 2.1. Materials

Table 1 shows the nominal chemical composition according to technical data from the International Nickel Company Inc. (New York, NY, USA) and energy-dispersive spectroscopy (EDS) analysis of the IN738LC specimens in this work, with the γ′-phase (Ni_3_(Al, Ti)) as the principal strengthening element in the γ-matrix formed by the balance of 65% Ni with Cr and Co [26].

### 2.2. Experimental Methods

**Figure 1:**
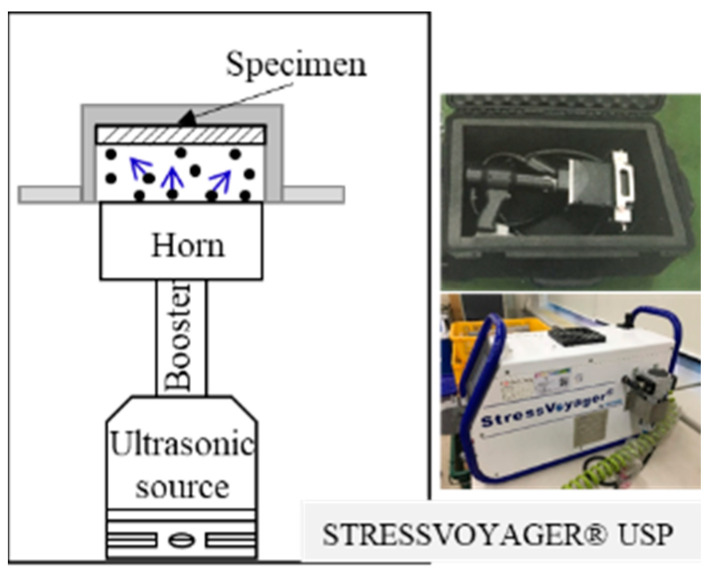
Ultrasonic shot peening (USP).

For USP (Figure 1), the experimental conditions were as follows (Table 2): treatment times, 20, 40, 50, 60, and 70 min; amplitude, 70 μm; ball diameter, 1.5 mm; frequency, approximately 20 kHz for stability.

**Figure 2:**
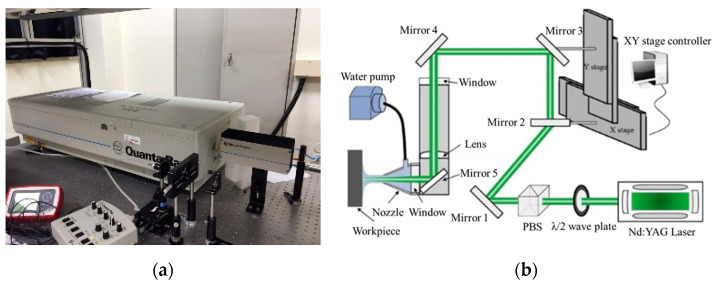
(**a**) The equipment of LSP; (**b**) Schematic diagram of the LSP setup [reprinted/adapted with permission from Ref. [27]; Copyright 2019, Elsevier].

For LSP (Figure 2), IN738LC plates were cut into 25 × 25 × 10 mm^3^ specimens. The peening specimens were milled prior to peening treatment to minimize defects and variation. Given the surface quality required for typical applications and to accurately assess the surface attributes and execute LSP uniformly, the surface was smoothened with a milling machine. All samples used for LSP were milled on the surface and then heat treated to reduce milling-induced stress [27]. The surface of each specimen was coated (wrapped) with a 100-μm-thick thermally protective layer of Al foil, which enhanced the laser absorption efficiency while preventing surface melting and damage [28]. Table 3 lists the LSP characteristics of the high-energy Nd: YAG laser (Spectra-Physics/Quanta-Ray Pro 350, Precision II-PL, Continuum, linearly polarized) that was utilized. The laser beam was focused along the centerline of a water jet impinging on the sample. The water prevented the evaporation of the specimen, and the resulting high-density vapor was rapidly ionized by inverse bremsstrahlung to produce metal plasma [29]. The power density ranged from 4 to 12 GW/cm^2^, the laser was concentrated to a 1-mm diameter spot, and multiple passes of the laser across the entire sample were used: 1, 2, 6, and 10 (referred to as 1, 2, 6, and 10 T, respectively). The overlap ratio was 50%, i.e., the overlap between two neighboring laser spots was set to 50% to achieve a homogeneous morphology [30].

The fatigue tests on the LSP specimen were conducted at 25 and 850 °C on low cycle fatigue (LCF) testing equipment (INSTRON 8861 ± 3.5 ton). To measure the fatigue strength at high temperatures (850 °C), fatigue specimens with a 6-mm diameter were exposed to tensile loading equipped with an 11.5-mm extensometer under strain control, capable of test speeds of 0.1%/s and under fully reversed loading, load ratio of R = −1. For each condition, the fatigue tests were repeated at least four times, and the average value was reported.

### 2.3. Microstructural Observation

A Digital Micro Vickers Hardness Tester from Future-Tech was used to measure the microhardness with 200 gf loads for 10 s dwell time. To estimate the microhardness depth profile, at least ten measurements were made and averaged with the error estimation. For the microstructural analyses using high-resolution scanning electron microscopy (HRSEM, JSM−7900F, (JEOL Ltd., Tokyo, Japan, equipped with EDS) and field-emission scanning electron microscopy (FESEM, Mira II LHM, Tescan, Brno-Kohoutovice, Czech Republic), the treated specimens were cut into two halves, and the cross-section was mounted and polished by automatic polisher with 1 μm diamond paste and then colloidal silica for 20 min. The specimen was electro-etched in a solution of phosphoric acid, sulfuric acid, and distilled water (4.7:1:2 ratio by volume) at a voltage of 2–4 V for approximately 15 s to clearly distinguish between γ′ and other phases. Image-Pro PLUS was used to measure the size of the precipitates. FESEM equipped with an EDAX, US/velocity electron backscatter diffraction (EBSD) camera was used for the microstructural characterization. EBSD analysis was performed to determine the grain orientations from the cross-sectional view and reveal the deformation depth from the peening surface. For transmission electron microscopy (TEM, FEI Ltd., Hillsboro, OR, USA, operated at 200 kV), the samples were ground to a thickness of ~100 μm using #600–#2000 grit SiC sandpapers and then electropolished using a Struers TenuPol−5 twin-jet polisher with a solution of 10% perchloric acid and 90% acetic acid at a current of 90–120 mA. For TEM observation of the cross-sectional view, two thin cross-sectional plates were polished down to ~100 μm and set vis-à-vis on the 3-mm single oval slot grid and electropolished [31]. A focused ion beam scanning electron microscope (FIB, Helios 450 F1; FEI Ltd., Hillsboro, OR, USA) was used for the preparation of the TEM specimen of the uppermost region. TEM analysis was performed using a Philips CM200FEG microscope (200 kV). Contact mode measurements were performed for atomic force microscopy (AFM, XE-100; Park Systems, Suwon, Korea) at a scan rate of 0.3 Hz to analyze the surface roughness. The 3D topographies of all peened samples were measured at a scanning length of 30 μm in ambient air.

## 3. Results and Discussion

### 3.1. Surface Roughness

**Figure 3:**
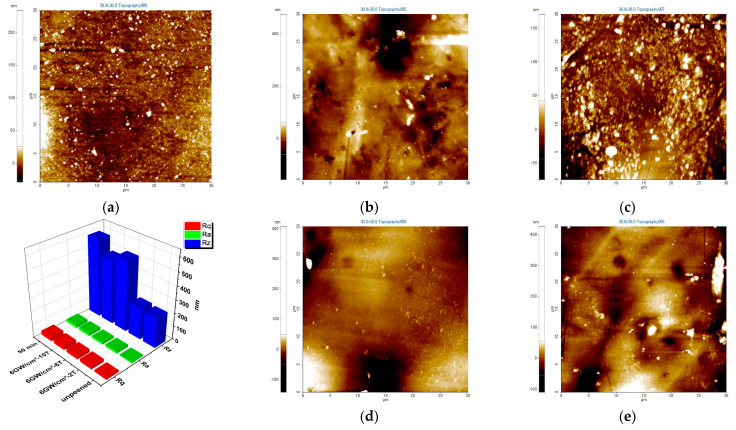
AFM surface roughness of the specimens: (**a**) unpeened, (**b**) USP: 50 min, LSP−6 GW/cm^2^ (**c**) 2 T, (**d**) 6 T, and (**e**) 10 T. The roughness parameters Rq, Ra and Rz are represented by red, green, and blue columns in the graph.

Figure 3a–d show the 3D surface topography of the specimens before and after USP and LSP. On the surface shown in Figure 3, the roughness parameters Rq (root mean square roughness), Ra (roughness average), and Rz (average maximum height of the profile) were measured [32]. Rq is the root mean square value of the deviation of the profile from the average line within the sampling length, which is the root mean square parameter corresponding to Ra. Ra is a commonly used parameter of roughness and is the arithmetic mean of the deviation of the profile from the average line defined within a sampling length. Rz is the maximum peak–valley height within the profile sampling length. The roughness values of USP (Rq = 32.0 nm; Ra = 21.0 nm; Rz = 588.0 nm) were greater than those of the unpeened specimen (Rq = 13.1 nm; Ra = 8.2 nm; Rz = 227.4 nm).

The graph shown in Figure 3 reveals that Ra increased with the number of laser peening passes: 2 T (Rq = 22.3 nm; Ra = 16.5 nm; Rz = 235.8 nm), 6 T (Rq = 25.0 nm; Ra = 16.6 nm; Rz = 529.0 nm), and 10 T (Rq = 28.2 nm; Ra = 20.0 nm; Rz = 485.5 nm) at 6 GW/cm^2^.

Specimens treated with USP exhibited even more severe surface roughness. Therefore, LSP is more suitable than USP for stabilizing surface integrity. With increasing LSP passes, the surface roughness (Ra) increased but was still less than that of the USP specimen.

### 3.2. Microhardness

**Figure 4:**
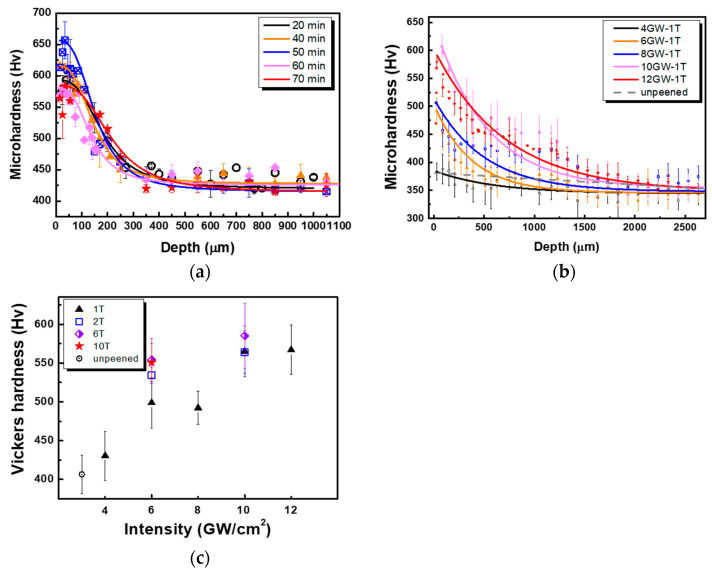
Microhardness variation upon peening treatment: (**a**) USP, (**b**) depth profile after one LSP pass, and (**c**) multiple LSP passes on the surface.

Figure 4a illustrates the microhardness profile of USP specimens during peening at various intervals. The microhardness reached a maximum after 50 min and subsequently decreased over the remaining treatment duration, although the impact depth remained almost constant at ~600 μm.

Figure 4b,c show the microhardness depth profiles and surface of LSP specimens using the indicated power density. A stronger hardness was achieved with a greater power density. The Vickers hardness generally increased with the power intensity. At 4 GW/cm^2^, the affective depth reached approximately 1000 μm, increasing to approximately 2500 μm at 12 GW/cm^2^, and the surface hardness of the specimen increased by 31.8% from 430 to 567 Hv. When the power density was 6 GW/cm^2^, the depth of the treated region increased with the number of peening passes. The specimen surface hardness increased by 10.2% from 499 Hv at 1 T to 550 Hv at 10 T. At 10 GW/cm^2^, the specimen surface hardness increased by 3.5% from 565 to 585 Hv as the number of the peening passes increased from 1 to 6 T.

The USP results show that the hardness was highest after peening for 50 min and then decreased with the peening time due to the recrystallization of γ and the recovery of cuboidal γ′. The deeper affected layer is the primary benefit of LSP over USP. The shock wave generated by LSP appears to transmit with less localized energy consumption, resulting in a more even distribution of defects such as dislocations. In addition, LSP can be readily carried out on less accessible areas of a surface that shot balls cannot reach and where improved fatigue resistance is most desired.

### 3.3. Microstructural Evolution

**Figure 5:**
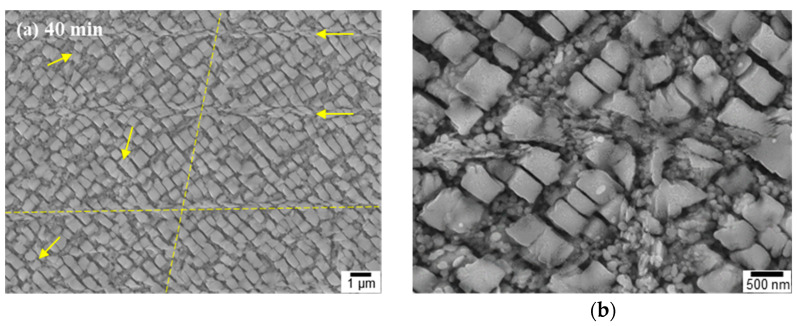

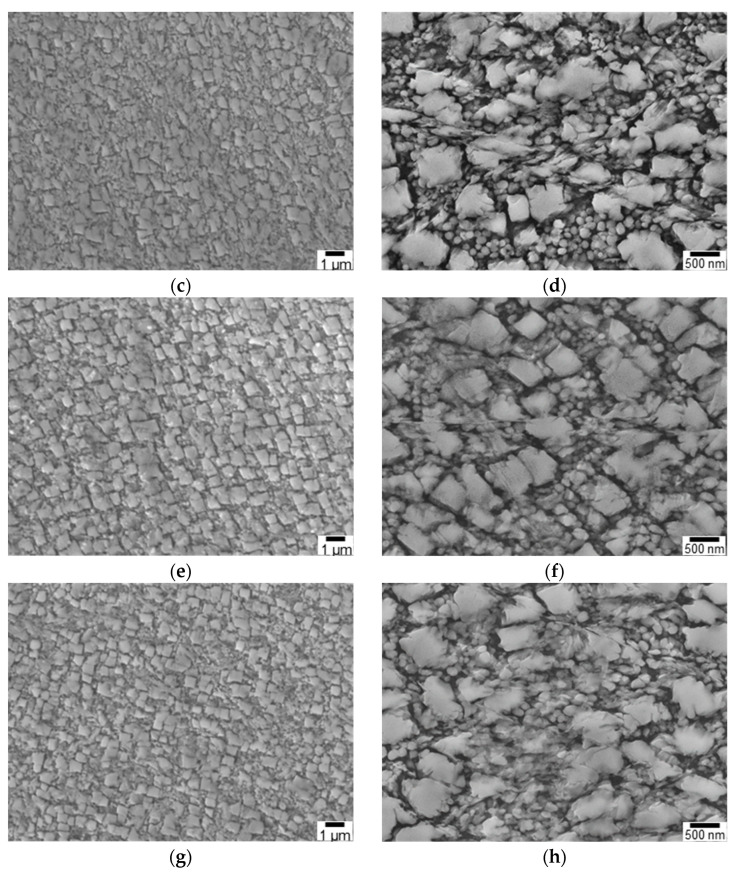
SEM images of shot-peened IN738LC recorded from the surface of the specimen, showing shearing across both γ′ and γ channels with peening time: (**a**,**b**) 40 min, (**c**,**d**) 50 min, (**e**,**f**) 60 min, and (**g**,**h**) 70 min (yellow line/arrow shows the path of shearing).

Figure 5 shows the micrographs of the USP-treated IN738LC alloy with longer treatment times. After USP, typical shear bands appeared on the surface layer with slightly deformed γ′ from the initial cuboidal shape (Figure 5a–h). In USP, the coarse γ′ broke down and the fine γ′ enlarged at the near-surface.

**Figure 6:**
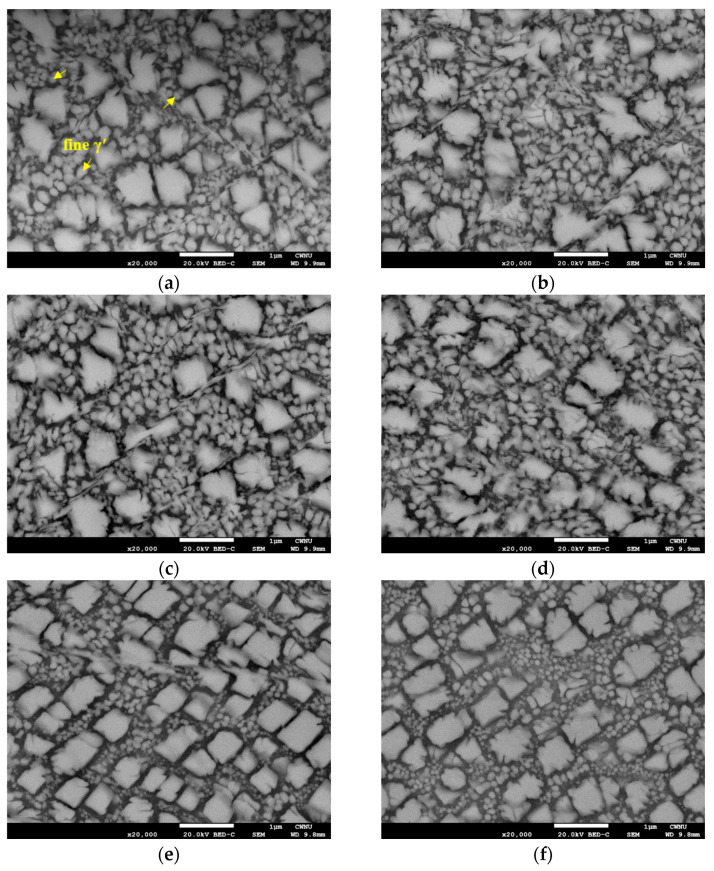
Typical SEM micrographs after 50 min USP at various depth: (**a**) ~5 μm, (**b**) ~10 μm, (**c**) ~20 μm, (**d**) ~50 μm, (**e**) ~100 μm, and (**f**) ~200 μm.

Figure 6 shows the cross-sectional image of the peening surface recorded ~200 μm from the surface. The microstructures of the deformed zone (≤100 μm) and the matrix (~200 μm) unaffected by USP are shown in Figure 6a–d. At a depth of approximately 100 nm from the peening surface, γ′ is deformed and elongated laterally from the shearing direction. Under high peening pressure, the cuboidal or rectangular γ′ phases developed a rhombohedral structure. The size of the γ′ precipitates increased with decreasing depth. At depths of 5, 20, 100, and 200 μm, the size of the fine γ′ precipitates was 0.15 ± 0.06 μm, 0.13 ± 0.05 μm, 0.10 ± 0.04 μm, and 0.07 ± 0.02 μm, respectively. Shear bands were also present, demonstrating the severity of alloy deformation upon USP. The microstructural changes at specific depths show that from deep below the surface, γ′ broke into smaller particles via high-density shearing.

**Figure 7:**
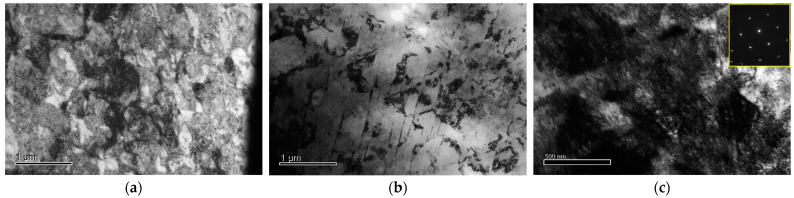
Typical TEM micrographs and corresponding selected area diffraction pattern (SADP) after 50 min USP at: (**a**) top, (**b**) ~20 μm, and (**c**) ~50 μm.

In the TEM micrograph (Figure 7) of the USP specimen after 50 min, a high shear band density and modified crystalline structure were observed. Furthermore, the grain refinement often observed in stainless steel treated with USP did not appear in this material (Figure 7c), partly due to the much higher hardness of the alloy.

**Figure 8:**
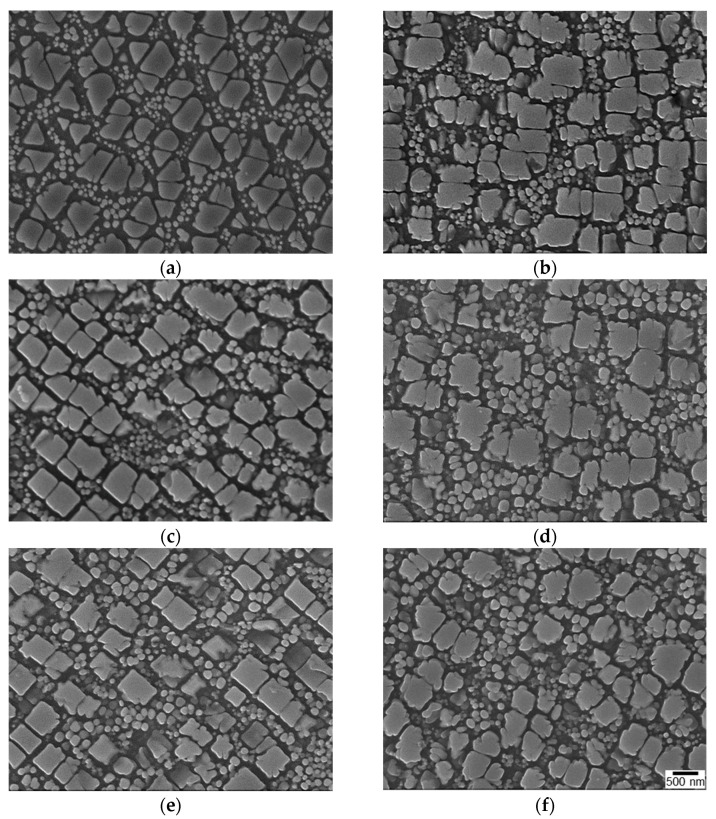
Surface SEM micrographs upon LSP with different laser power densities: (**a**) unpeened, (**b**) 4 GW/cm^2^, (**c**) 6 GW/cm^2^, (**d**) 8 GW/cm^2^, (**e**) 10 GW/cm^2^, and (**f**) 12 GW/cm^2^.

**Figure 9:**
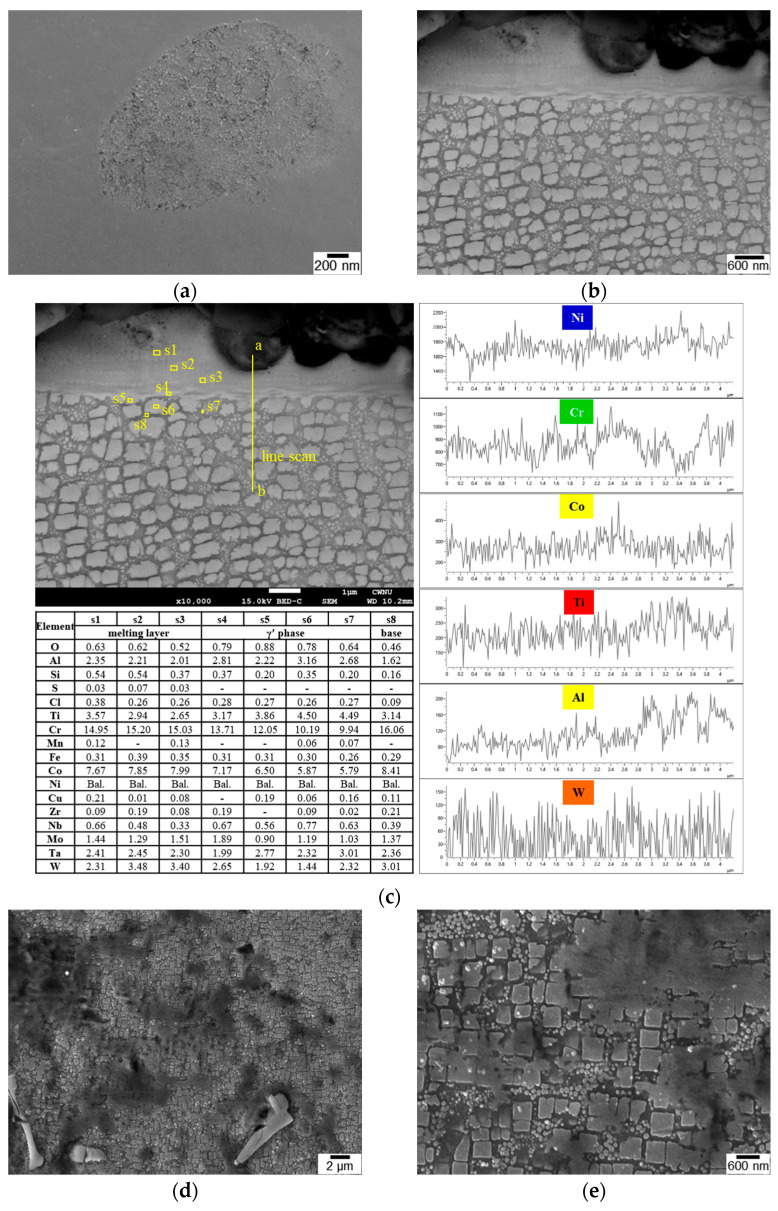
SEM analyses of the LSP specimens: (**a**) plan view, (**b**) cross-section BSE image, (**c**) EDS point analysis (Table) and line profile of the 6 GW/cm^2^−10 T specimen, and (**d**,**e**) low and high magnification image of the 10 GW/cm^2^−10 T.

Figure 8 shows SEM images of the surface microstructure before and after LSP. γ matrix channels surrounded the γ′ precipitates. The SEM images revealed a specimen surface with deformed areas that did not melt following LSP (Figure 8b–f). Even though an Al foil coating was covering the specimen, thermal damage was observed as a result of the interaction between the laser and the surface (Figure 9). As the surface was heated above its melting point, molten γ′ flow at the top reached about 20 nm in depth with increasing surface roughness. The melting point of the IN738LC alloy was 1325 °C [33], which was much lower than the temperature of the plasma formed near the surface [34,35,36,37,38]. Considering the microhardness, which is related to its strength, and the appearance of the thermal damage on the surface, 10 GW/cm^2^ and 1T were regarded as the optimum processing conditions.

**Figure 10:**
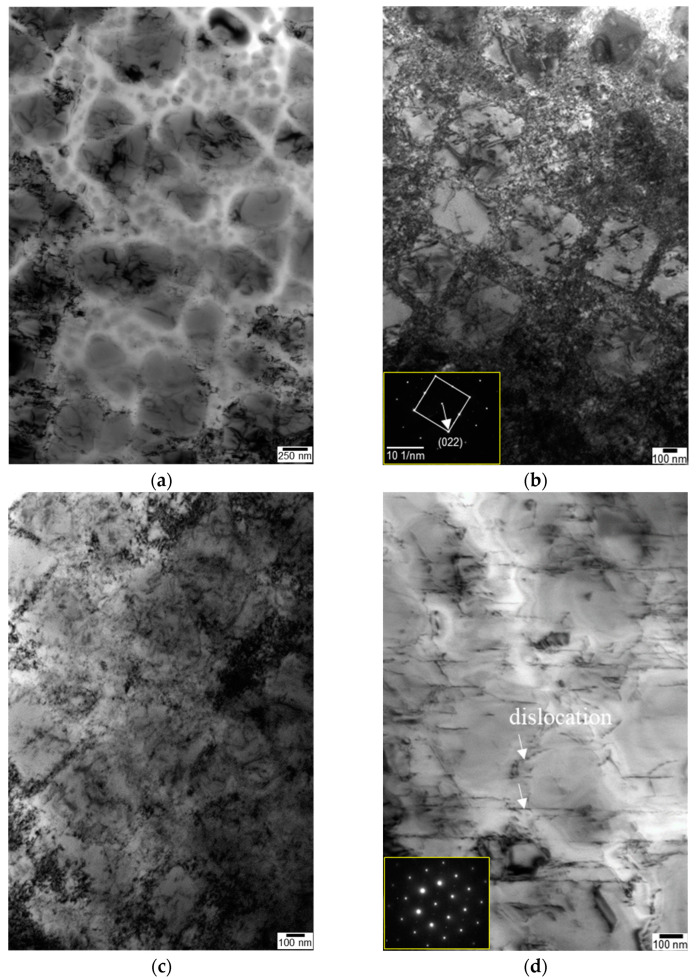
Typical TEM micrographs and corresponding SADP of the topmost region of the specimen: (**a**) unpeened, (**b**) 6 GW/cm^2^−10 T, (**c**) 10 GW/cm^2^−10 T, and (**d**) 12 GW/cm^2^−1 T.

The pressure of the laser shock wave induced plastic deformation and dislocations, resulting in the change of shape and size of γ′ as well as changes in dislocation multiplication (Figure 10). When the laser power density was increased from 4 to 12 GW/cm^2^, the size of fine γ′ increased from 0.13 to 0.17 μm and the area fraction of fine γ′ increased from roughly 8% to 18%. The dislocation density also increased (Figure 10), which is consistent with the microhardness analysis. Higher power density and more peening passes generated multiple dislocations and some dislocation entanglements in the γ matrix and γ′ phase.

Figure 10 shows the TEM micrographs of the LSP-treated specimen at a power density of 6 GW/cm^2^. As the number of passes increased, the modification of the microstructure became more severe. The γ′ phase was cut off in several places by dislocations, but no shear bands were observed. Figure 10b shows the dislocations trapped in and around the γ′ phases, without shear bands.

The dislocation density introduced by LSP increased with the laser power density to a certain point. Then, the local dislocation density decreased as the power density increased further (Figure 10).

**Figure 11:**
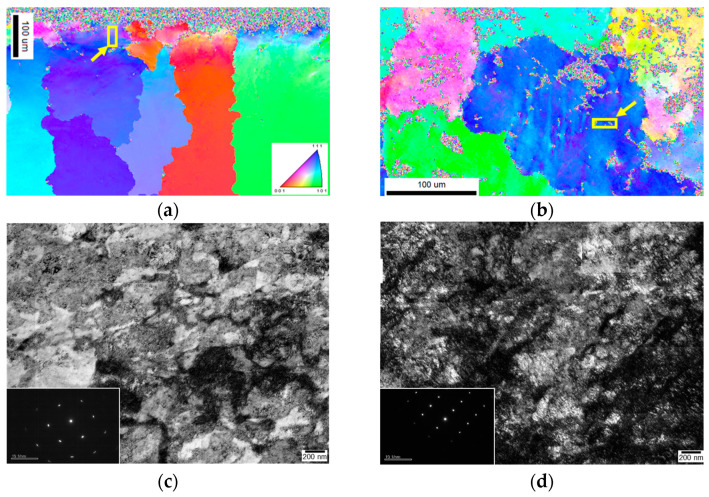
Typical TEM micrographs and corresponding SADP of the top surface of (**a**,**c**) USP−50 min, and (**b**,**d**) LSP−6 GW/cm^2^−10 T specimen.

Figure 11a,b show the position of the target grains (orientation) of the specimen after treatment by USP and LSP as determined by EBSD analysis, and the area shown in the yellow box represents the location of the TEM specimen prepared by FIB. Figure 11a shows the cross-sectional view of the specimen after treatment by USP. Figure 11b shows the surface view of the specimen after treatment by LSP. The top surface was the position selected for both approaches. Figure 11c,d show the TEM analysis of the microstructure of the top region of the specimen after treatment by USP and LSP. USP produced shear bands and localized dislocations, while LSP showed only dislocations distributed throughout the entire TEM foil. The diffraction patterns taken at the same sample depths show that USP introduced more deformations than LSP. Thus, shear bands and dislocations doubly affected the strengthening of the specimen, which resulted in a greater increase in hardness.

High strain rate and deformation upon USP introduced considerable shear to the precipitate (γ′) in the alloy, leading to intensive strengthening. When the peening time was increased above 60 min, the recovery of the shape of coarse γ′ and the coalescence of coarse and fine γ′ resulted in a slightly smaller increase in microhardness. While the shots impacted the overall specimen multiple times, laser beams scanned over the specimen once with overlap at the edges. Thus, the overall impact energy absorbed by the specimen for LSP was much lower. Additionally, the deformation and the dislocation density of the LSP specimen were much lower and relatively uniform throughout the treated surface.

Upon USP treatment, microstructural modification and increased hardness localized to the surface region were observed, whereas a relatively low dislocation density uniformly distributed throughout the specimen in a much deeper region occurred in the LSP specimen. These observations can be attributed to the multidirectional and multiple impact nature of USP and the uniform single path scanning of high-energy LSP.

**Figure 12:**
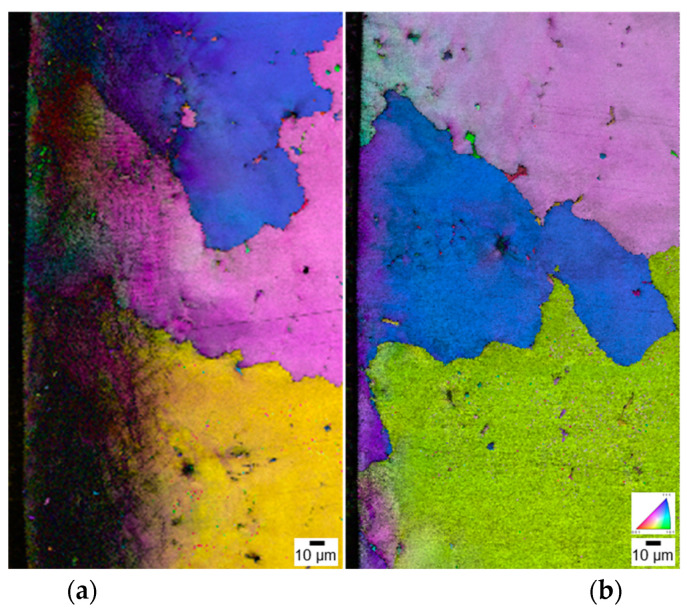
EBSD image quality maps and inverse pole figures showing typical microstructures at the top surface of (**a**) USP−50 min with sub-grain boundaries, and (**b**) LSP−6 GW/cm^2^−10 T specimen.

The EBSD results show image quality maps and inverse pole figures with grain orientations (Figure 12). The USP-treated specimen exhibited grain deformation and low-angle grain boundaries (2°–15°, black slip lines in Figure 12a) focused on the top layer (~100 μm). However, no low-angle grain boundaries were created on the surface of the LSP-treated specimen, demonstrating the grain refinement of USP on the surface.

### 3.4. Fatigue Test

**Figure 13:**
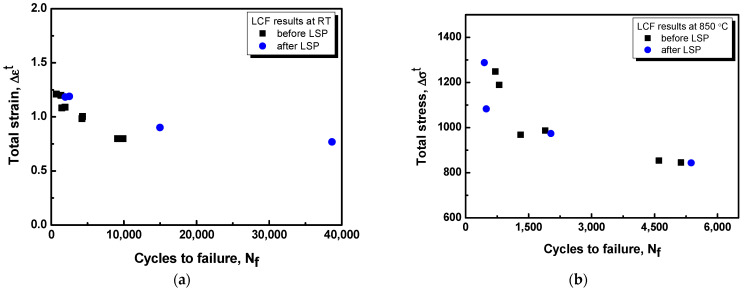
Stress-life (S-N) curves of fatigue test before and after LSP at (**a**) room temperature (25 °C) and (**b**) high temperature (850 °C).

Figure 13 shows the stress-life (S-N) fatigue behavior of IN738LC at room temperature (25 °C) and high temperature (850 °C) in terms of the total strain/stress as a function of the number of cycles until failure (at R = −1). The fatigue strength of specimens before peening at high temperatures (850 °C) was lower than that at room temperature (25 °C), and the fatigue endurance strength after 5000 cycles decreased by approximately 33%. The fatigue strength of the specimen after LSP at room temperature increased by approximately 18% compared to that before LSP after 10,000 cycles. With a slight increase in LCF at room temperature, the positive effects of LSP are sustained. The rapid stress relaxation was caused by the decrease in dislocation density at high temperatures [39]. Thus, a large dislocation density caused by LSP is insufficient to maintain high-temperature fatigue life.

## 4. Conclusions

The microhardness and microstructure of the surface layer after the USP and LSP treatment of IN738LC were investigated herein. The following details were considered: (1) surface modification, (2) microstructural modification, (3) mechanical properties and optimum process condition, (4) mechanism of mechanical property improvement, and (5) respective application of USP and LSP:

1. Surface modification: The surface roughness (unpeened specimen: Rq = 13.1 nm) increased after peening treatment due to the greater intensity of plastic deformation. The roughness of the USP specimens (Rq = 32.0 nm) was greater than that of the LSP specimens (Rq = 28.2 nm).
2. Microstructural modification: USP resulted in more localized deformations and microstructural fluctuations than LSP. The size of fine γ′ on the uppermost region increased by ~114% from 0.07 to 0.15 μm. Thus, energy was absorbed in the impacts of the shot balls generating high-density entangled dislocation structures and shear bands. Compared to USP treatment, the size of γ′ increased by ~31% and remained cuboidal after LSP, which helped increase the creep resistance of the surface. LSP showed fewer defects with an even distribution, such as high-density planar dislocations but without shearing.
3. Optimum process condition: USP for 50 min at an amplitude of 70 μm, 20 kHz frequency, and 1.5 mm ball size (SUJ2) were the optimum conditions, with an effective depth as low as ~600 μm from the peening surface, yielding a surface hardness of ~650 Hv. LSP provided optimum results at 10 GW/cm^2^, achieving a hardness of ~630 Hv and an effective depth of ~2500 μm. High power density (12 GW/cm^2^) or multiple passes at 10 GW/cm^2^ or 10 T at 6 GW/cm^2^ caused either lower hardness or thermal damage to the treated surface.
4. Improved mechanical properties: Severe shearing localized in the surface region by USP led to sub-grain boundary formation and premature creep rupture. Thus, USP can be used to strengthen components for use as cast forms, since the introduced grain boundaries of orientation were sufficient to improve the rupture life. In contrast, LSP, which does not produce low-angle grain boundaries, can improve single-crystal forms much more effectively than USP due to the sensitivity of the creep life to the presence of grain boundaries.
5. Strengthening mechanism and application: Before LSP, the fatigue resistance of IN738LC was greater at 850 °C than it was at room temperature. LSP improved the fatigue life of the material at room temperature, whereas stress relaxation occurred at high temperatures (850 °C). LSP treatment and the defects generated thereby did not seem to strengthen or improve the fatigue properties sufficiently to extend fatigue life at high temperatures. Thus, LSP may be more appropriate for components used in lower temperature applications requiring high strength and large grains.

## Figures and Tables

**Table 1:** Chemical composition of IN738LC superalloy (wt.%).

	Cr	Co	Ti	Al	W	Mo	Ta	Nb	Fe	Si	Mn	C	Cu	Zr	B	Ni
Ref.	15.7–16.3	8.0–9.0	3.2–3.7	3.2–3.7	2.4–2.8	1.5–2.0	1.5–2.0	0.6–1.1	≤0.35	≤0.3	≤0.2	0.09–0.13	≤0.1	0.03–0.08	0.007–0.012	Bal.
EDS results	15.53	7.89	2.76	3.22	3.82	1.65	1.53	0.40	0.09	−	0.03	−	0.31	0.11	−	Bal.

**Table 2:** USP parameters.

Treat. Time	Amplitude	Diameter of Ball	Number of Balls	Frequency
20	70 μm	1.5 mm	393 pieces	20 kHz
40				
50				
60				
70				

**Table 3:** LSP parameters in this study.

Laser	Wavelength	Pulse Energy	Pulse Duration	Repetition Rate	Spot Size	Power Density	Overlap	Peening Passes	Confinement Media
Nd: YAG	532 nm	≤1.4 J	8 ns	10 Hz	1 mm	4, 6, 8, 10, 12 GW/cm^2^	50%	1, 2, 6, 10 T	water

## Data Availability

Not applicable.
